# Effect of emotional stimulus on response inhibition in people with mild cognitive impairment: an event-related potential study

**DOI:** 10.3389/fnins.2024.1357435

**Published:** 2024-04-30

**Authors:** Jing Wang, Cheng Li, Xiaohong Yu, Yue Zhao, Enfang Shan, Ying Xing, Xianwen Li

**Affiliations:** School of Nursing, Nanjing Medical University, Nanjing, Jiangsu, China

**Keywords:** emotion, response inhibition, mild cognitive impairment, event-related potential, N2, P3

## Abstract

**Background:**

A few studies are emerging to explore the issue of how aging promotes emotional response inhibition. However, there is a lack of empirical study concerning the impact of pathological cognitive impairment on emotional response inhibition. The present study investigated the effect of emotion on response inhibition in people with mild cognitive impairment, the stage of cognitive impairment before dementia.

**Methods:**

We used two emotional stop-signal tasks to explore whether the dual competition framework considering limited cognitive resources could explain the relationship between emotion and response inhibition in mild cognitive impairment.

**Results:**

The results showed that negative emotions prolonged N2 latency. The Go trial accuracy was reduced in the high-arousal negative conditions and the stop-signal reaction time was prolonged under high-arousal conditions. This study also verified impaired response inhibition in mild cognitive impairment and found that negative emotions prolonged P3 latency in mild cognitive impairment.

**Conclusion:**

Emotional information interferes with response inhibition in mild cognitive impairment populations, possibly because emotional information captures more attentional resources, thus interfering with response inhibition that relies on common-pool resources.

## 1 Introduction

Emotion processing is integral to daily life and impacts various cognitive abilities, especially executive functioning (Prehn et al., 2011). Inhibitory control is a crucial component of executive function, preventing access to irrelevant information, clearing irrelevant behavior, and reducing interference deriving from competing distractions (Friedman and Miyake, 2004). Inhibitory control is essential for regulating everyday behavior and for effective adaptation in complex situations (Griffith et al., 2003; Hsieh et al., 2016). Given the daily relevance of emotion processing and response inhibition, clarifying how these constructs interact and what influences them is crucial.

Existing studies focused on comparing older and young adults to explore the role of aging in influencing the process of emotion and response inhibition. Studies utilizing the emotional Stroop task, Stop-Signal task and Go/No-Go task found that healthy older adults with positive emotions had greater emotional response inhibition than younger adults (Waring et al., 2019; Williams et al., 2020; Almdahl et al., 2021), and task-relevant happy information enhanced response inhibition in older adults. The older adults with pathologic cognitive impairment were not included in these studies. However, many older adults are now experiencing pathological cognitive decline beyond the normal rate of aging (Langa and Levine, 2014; Toepper, 2017). The stage before pathological cognitive decline develops into dementia is known as mild cognitive impairment (MCI). The annual conversion rate of MCI to dementia is 10–15%, increasing to 80–90% after about 6 years, and the annual conversion rate of dementia in normal people is only 1–2% (Gabryelewicz et al., 2007; Eshkoor et al., 2015). Based on this, MCI is at greater risk for conversion to dementia and is a crucial population for the prevention of dementia, so the impact of pathologically impaired cognitive functioning on the control of emotional inhibition also deserves our attention.

Previous studies exploring the effects of aging on emotional response inhibition have examined the effects of emotion on inhibitory control from the perspective of valence (Waring et al., 2019). As it turned out that emotional response inhibition for positive information is enhanced (Williams et al., 2020). Emotional facilitation was also found in a population with aggressive psychiatric disorders. The authors explained this result as emotional information modulating motor responses by activating occipital brain regions (Pawliczek et al., 2013). However, some studies present a different opinion, such as the Krakowski study, which found that negative affective triggers disrupted response in schizophrenic patients with cognitive deficits (Krakowski et al., 2016). It is worth noting that the above studies were conducted only regarding the valence dimension. According to the classical Dimensional Model of Emotion, emotions are characterized by two dimensions of valence and arousal (Shankman and Klein, 2003). Some studies confirmed that while emotional information affects response inhibition, there was no difference in positivity or negativity (Zhang and Lu, 2012; Zhao et al., 2019). The arousal values between positive and negative pictures were higher than neutral ones. This implies that the effect of emotion on inhibition mainly comes from high arousal characteristics. Based on the above information, emotion still influences inhibitory control in pathological cognitive decline, but emotion’s function is unclear. In the present study we further clarify the role of emotions in facilitating or interfering with inhibitory control in MCI.

Some studies have confirmed that emotion regulation is impaired in MCI (Bora and Yener, 2017; Liu et al., 2022), mainly focusing on negative emotions and that the recognition of positive faces, such as joy, happiness, and hope, is no different from healthy older adults (McCade et al., 2013; Elferink et al., 2015; Barbieri et al., 2022). This seems to suggest that MCI retains the “positive effect.” In addition, based on scholars’ proposal of the dual competition models, it is known that emotions and cognitions compete with each other, and the process largely depends on attentional capture (Pessoa, 2009). MCI often have a negative bias due to impaired emotion regulation. Regarding emotional memory, MCI tends to recall more negative words and has negative emotional memory biases (Mah et al., 2017), suggesting that negative stimuli will capture more of the attention of MCI and be challenging to disengage. Furthermore, studies have shown that experiencing depression is very common in MCI. A meta-study showing the overall pooled prevalence of depression in MCI was 32%, with the hospital samples reaching 40% (Ismail et al., 2017). Depressive symptoms further exacerbate impaired emotion regulation, focusing more attention on negative information and making it harder to disengage (Weiss et al., 2008). High arousal stimuli with attention-grabbing features based on the above claim make it difficult for people with MCI to disengage (Mather and Sutherland, 2011). Consequently, we hypothesize that low arousal positive information promotes the inhibitory control of MCI, and high arousal negative and high arousal positive information will interfere with inhibitory control.

An increasing number of studies have confirmed that response inhibition is a set of processes rather than a unitary process (Pires et al., 2014). The rapidity of inhibitory control behavior makes event-related potential (ERP) technology with high temporal resolution widely used to study the time course involved in response inhibition (Plawecki et al., 2018; Chen et al., 2019; Lee and Kang, 2020). Since action-stopping is challenging to quantify directly, the stop-signal task was chosen to quantify inhibition by estimating the latency of cessation (stop-signal reaction time, SSRT; Boehler et al., 2010). The Stop-signal task paradigm with non-emotional stimuli has consistently identified N2 and P3 related to temporal features of response inhibition (Kok et al., 2004). N2 (200-400ms) reflects attentional control and conflict resolution (Folstein and Van Petten, 2008; Zhang and Lu, 2012). P3 (300-600ms) reflects motor inhibition processes and inhibitory performance evaluation (Falkenstein et al., 1995; Smith et al., 2008). Incorporating emotional stimuli into the stop-signal task provides new insights into exploring processes related to emotionally influenced inhibition (Stockdale et al., 2020). Existing studies provided some ERP evidence of emotional inhibitory control, such as that N2 or P3 amplitudes were typically reduced in people with emotion disorders. Especially for depressed patients, the study has shown that the NoGo-N2 component was reduced for positive images, while the NoGo-P3 component was reduced for emotional images compared to neutral images (Camfield et al., 2018). However, some studies have pointed out that depression and borderline personality disorders only have damage in the previous stage of inhibitory control (differ only in N2 amplitudes; Palmwood et al., 2017; Yang et al., 2021). Patients with significant cognitive impairment, such as schizophrenia, were often affected by the P3 component. The study found that for stop trials, a smaller P3 amplitude was found in the angry condition than in the neutral condition (Jia et al., 2023). As a result, while the ERP components elicited by the emotional stop signal task in different characterized populations were concentrated in N2 and P3, the conclusions on the timing of emotional influence on inhibitory control proceeded inconsistently. In the face of MCI with impaired cognition and emotion regulation, existing research has focused only on the stages of impairment of their inhibitory control. For example, Cid-Fernández et al. suggested that MCI showed poorer execution and smaller Go-N2 and NoGo-N2 amplitudes than healthy adults, whereas P3 amplitudes and N2 and P3 latencies did not differ between the groups (Cid-Fernández et al., 2014). This suggested that MCI was mainly reflected by the difficulty in conflict assessment rather than the speed of stop signal perception. Nevertheless, we do not know anything about the time course of emotional influences on inhibitory control in MCI.

In the present study, we used the stop-signal task from a neurophysiological perspective to investigate the effects and the temporal course of emotion on the inhibitory control of MCI to provide more details and timing of cognitive emotion regulation strategies for people with MCI. Specifically, Experiment 1 explores the effects of emotional valence on response inhibition, and Experiment 2 explores the effects of emotional arousal on response inhibition, and clarifies whether emotional arousal has a moderating effect on valence. In addition, we also assessed participants’ performance on several neuropsychological tests to evaluate potential relationships between the performance of inhibitory control behaviors and electrophysiological measures and neural indices in MCI.

## 2 Materials and methods

### 2.1 Study participants

Forty-four participants from the senior day center and nursing home in Nanjing, China, were recruited to participate in two experiments (Experiment 1 and Experiment 2), and all gave written informed consent. The study adhered to the Declaration of Helsinki and was approved by the Ethics Committee of Nanjing Medical University, Ethics Approval Number: (2021) 553.

Each participant underwent complete data collection, scale assessment including the Beijing version of the Montreal Cognitive Assessment (MoCA; Yu et al., 2012), Clinical Dementia Rating (CDR; Morris, 1993), Activities of Daily Living (ADL; Katz et al., 1963), Trail Making Test A (TMT-A; Llinàs-Reglà et al., 2017) and experimental tasks. Participants with MCI were diagnosed based on the Petersen’s criteria (P-MCI; Petersen et al., 1999), including a memory complaint verified by an informant; isolated memory impairment on neuropsychological testing and general cognitive function preserved; normal activities of daily living; not meeting criteria for a diagnosis of dementia. It is well known that demographic variables can influence the validity of cognitive screening tools. We grouped according to the MOCA optimal cutoff points of the Chinese elderly studied by Lu et al. (2011). Participants in both groups were right-handed, and excluded from the study were patients with severe hearing and visual impairments; psychotropic drugs, alcohol dependence; anxiety disorders, depression, schizophrenia, and other serious mental illnesses; encephalitis, traumatic brain injury, and other organic brain diseases.

### 2.2 Material

The stimulus material was selected from the International Affective Picture System (IAPS) database (Bradley and Lang, 2007). Considering the cultural differences, we invited 20 participants over 50 from the Nanjing nursing institution to apply the Likert 9-point scale to rate the valence and arousal of 221 pictures from the IAPS (Huang et al., 2015). Based on the results of the picture ratings, a repeated measures ANOVA was used to select 20 positive pictures (valence: M = 6.358, SD = 0.169; arousal: M = 4.647, SD = 0.332), 20 neutral pictures (valence: M = 4.698, SD = 0.139; arousal: M = 4.533, SD = 0.295), and 20 negative pictures (valence: M = 3.128, SD = 0.167; arousal: M = 4.608, SD = 0.308), which served as emotional stimulus materials for Experiment 1. The main effect of emotional valence (*F* = 101.201, *p* < 0.001) was statistically significant, and the main effect of arousal (*F* = 0.147, *p* = 0.809) was not statistically significant. There was a significant difference in valence between the positive, neutral, and negative pictures, while the difference in arousal was not significant.

Based on the results of the picture ratings, a repeated measures ANOVA was used to select 20 low arousal/positive (valence: M = 6.350, SD = 0.176; arousal: M = 4.608, SD = 0.355), 20 high arousal/positive (valence: M = 6.510, SD = 0.219, arousal: M = 5.945, SD = 0.370), 20 low arousal/negative (valence: M = 3.093, SD = 0.176, arousal: M = 4.745, SD = 0.325), and 20 high arousal/negative (valence: M = 3.008, SD = 0.140, arousal: M = 6.025, SD = 0.254), which served as emotional stimulus materials for Experiment 2. The main effect of emotional valence (*F* = 118.728, *p* < 0.001) and the main effect of arousal (*F* = 16.780, *p* < 0.001) were statistically significant. Among the emotional pictures in Experiment 2, 30 were used in Experiment 1.

### 2.3 Procedures

**Figure 1:**
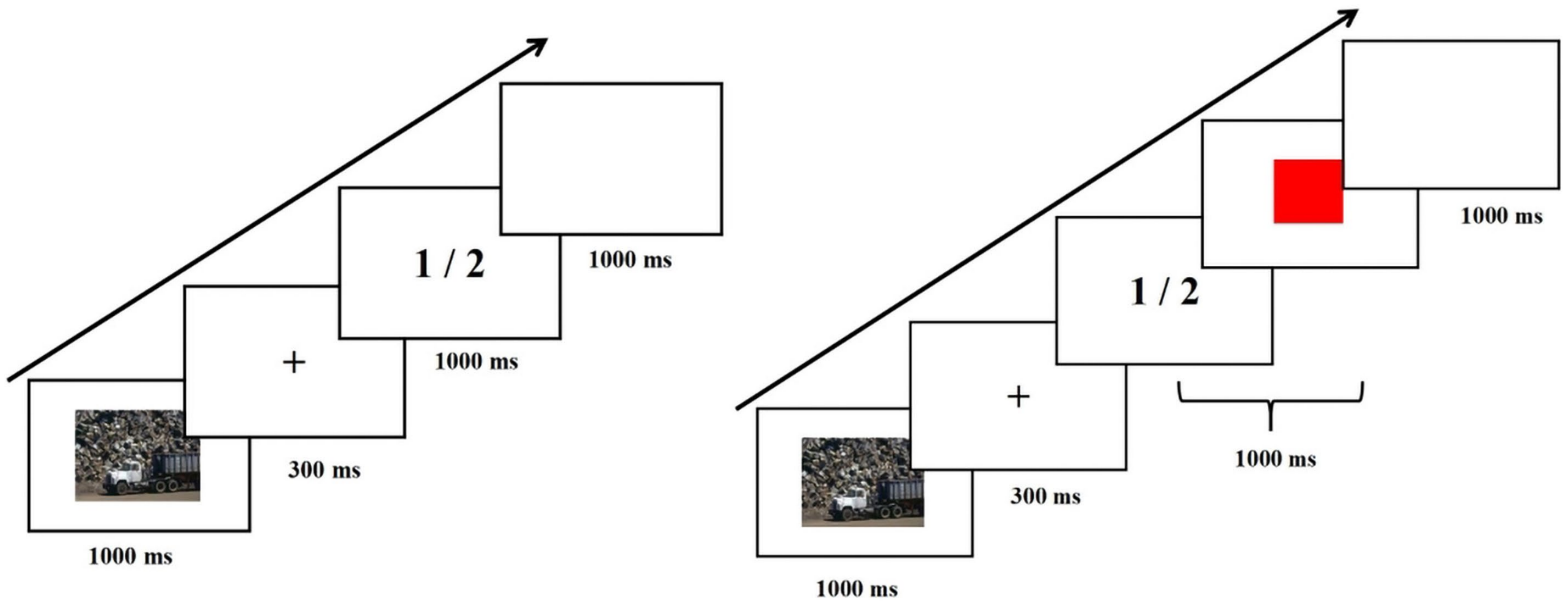
Flow diagram of stop-signal task.

The experiment presented the task process through E-prime software 3.0. We used an emotional stop-signal task to assess inhibitory control of emotional stimuli (Pawliczek et al., 2013), as shown in Figure 1. The stop-signal task contained go trials and stop trials, with 25% stop trials. Experiment 1 contained 270 go trials and 90 stop trials, which took about 20 min. Experiment 2 contained 360 go trials and 120 stop trials, which took about 27 min. The process of go trials is as follows: first, the emotional picture appears (1,000 ms), then the fixation point “+” appears (300 ms), then “1” or “2” (go stimulus) appears (1,000 ms), and finally, a blank screen appears (1,000 ms). Emotional stimuli are presented in a pseudo-random order. The go trials required participants to press the “F” key when “1” appeared and the “J” key when “2” appeared. The go stimulus disappeared immediately when participants pressed the key and disappeared after 1,000 ms if they did not. The only difference between the stop trials and the go trials is that a red square (stop signal) appeared after a “1” or “2” (go stimulus), and participants were asked to see the stop signal and not to respond with any key presses. Go stimulus and stop signal co-presentation time of 1,000 ms. The delay between the go stimulus and stop signals (Stop Signal Delay (SSD)) changed through the experiment in a staircase dynamic-tracking manner (50–800 ms), depending on the participant’s performance in the preceding stop trial (Berger et al., 2013). The stop signal disappeared immediately when the participant responded with a key press and disappeared after (1000-SSD) ms when no key presses were presented. Stop Signal Reaction Time (SSRT), which represents the time required for a stopping response, was calculated as the difference between the averaged RT to the go signal and the averaged SSD (Alyagon et al., 2020). Participants had a practice phase before conducting the formal experiment, and explanations were given to them to ensure they understood the task. Two experiments were conducted on the same day. Experiment 1 was always in first place, and Experiment 2 was in second place. There was a 30-min washout period between the two experiments.

### 2.4 EEG recording and analysis

The electroencephalogram (EEG) was recorded by the NeuroScan SynAmps 2 amplifier and a 64-channel Quik-Cap with Ag/AgCl sintered electrodes, placing them according to the 10–20 system. We ensured that all electrode impedances were below 5 KΩ at the beginning of the recording. All data was recorded in AC mode and was recorded with a band pass filter of 0.05–30 Hz, and sampled at a rate of 1,000 Hz. The pre-processing of EEG data is performed using the following steps: (1) EEG data were re-referenced to the average of two mastoid electrodes; (2) Selecting Constant for baseline correction; (3) Selecting Bandpass Filter and choosing the 30 Hz low-pass filter; (4) Ocular artifact removal threshold set to ±200 μV based on VEOG; Removing Bad Blocks with amplitude more than ±100 μV; (5) Segmenting, superimposing, and averaging the EEG data according to the event mark; Determining the time intervals for inhibitory component analysis as −200 ~ 800 ms (Pires et al., 2014); The number of trials used for ERP averaging under different emotional conditions was 30; (6) Selecting Pretrigger for baseline correction again. Based on previous studies on inhibitory control and the average ERPs waveforms in our study, we selected electrodes such as Fz, FCz, Cz, and CPz to analyze the N2 (200-320ms) and P3 (300-460ms; Pires et al., 2014; Carbine et al., 2018; Allen et al., 2022; Wang et al., 2022). The N2 (200-320ms) and P3 (300-460ms) amplitude and latency for each participant were obtained by segmenting, superimposing, and averaging the EEG data for each participant based on event markers.

### 2.5 Statistical analysis

SPSS 27.0 was used for statistical processing. We test the normal distribution of the sociodemographic and neuropsychological data firstly. And data were analyzed using *t*-tests if they conformed to the normal distribution, otherwise, the non-parametric tests were used. For gender, we use the chi-square test. In Experiments 1 and 2, behavioral and EEG data were analyzed using the repeated measures ANOVAs, including the accuracy of go trials and stop trials, SSD, SSRT, the mean amplitudes and latency of N2, and P3. All post-hoc tests were conducted using Bonferroni with appropriate corrections for multiple comparisons. The accuracy of stop trials in each experiment refers to the proportion of the number of correct responses in the stop trial to the total number of stop trials. The accuracy of go trials refers to the proportion of the number of correct responses in the go trials to the total number of go trials.

## 3 Results

### 3.1 Experiment 1

#### 3.1.1 Participant characteristics

Among the 44 participants, 8 were excluded because the collected ERP data had too many bad segments and could not be used, and 36 participants were eventually included, 18 in the MCI group and 18 in the health group. As can be seen in Table 1, the two groups did not differ in age, gender and years of education. The total score of MoCA in the MCI group was lower than that in the health group (*t* = 6.207, *p* < 0.001), and the cognitive function of the MCI group was decreased to different degrees, which were manifested in Visuospatial/Executive (*p* = 0.015), Naming (*p* < 0.001), Attention (*p* = 0.008), Language (*p* = 0.017), and Delayed recall (*p* = 0.005). Scores in both the health group and the MCI group were not statistically significant in the TMT-A.

**Table 1:** Sociodemographic and neuropsychological data for two groups in Experiment 1.

	The health group (*n* = 18)	The MCI group (*n* = 18)	χ^2^ /t/Z	*p*
Gender (Male / Female)	7 / 11	5 / 13	0.50	0.480
Age	55.50 (52.00, 61.00)	59.00 (57.00, 62.00)	−1.92	0.055
Education (years)	8.78 ± 2.82	8.17 ± 2.94	0.64	0.528
The total score of MoCA	25.61 ± 2.06	20.61 ± 2.73	6.21	**<0.001**
Visuospatial/Executive	4.00 (3.00, 5.00)	3.00 (2.00, 4.00)	−2.44	**0.015**
Naming	3.00 (2.00, 3.00)	1.50 (1.00, 2.00)	−3.37	**<0.001**
Attention	6.00 (6.00, 6.00)	5.00 (4.00, 6.00)	−2.67	**0.008**
Language	3.00 (2.00, 3.00)	2.00 (1.75, 3.00)	−2.39	**0.017**
Abstraction	1.00 (0.00, 2.00)	0.00 (0.00, 1.00)	−1.67	0.094
Delayed recall	3.06 ± 1.26	1.83 ± 1.20	2.98	**0.005**
Orientation	6.00 (6.00, 6.00)	6.00 (5.00, 6.00)	−1.25	0.213
TMT-A (errors)	0.00 (0.00, 0.00)	0.00 (0.00, 0.00)	−1.78	0.074
TMT-A (s)	50.50 (36.75, 68.00)	59.50 (46.00, 76.25)	−1.28	0.200

MoCA, the Montreal Cognitive Assessment; TMT-A, Trail Making Test A. All significant *p* values are shown in bold.

#### 3.1.2 Behavioral results

**Figure 2:**
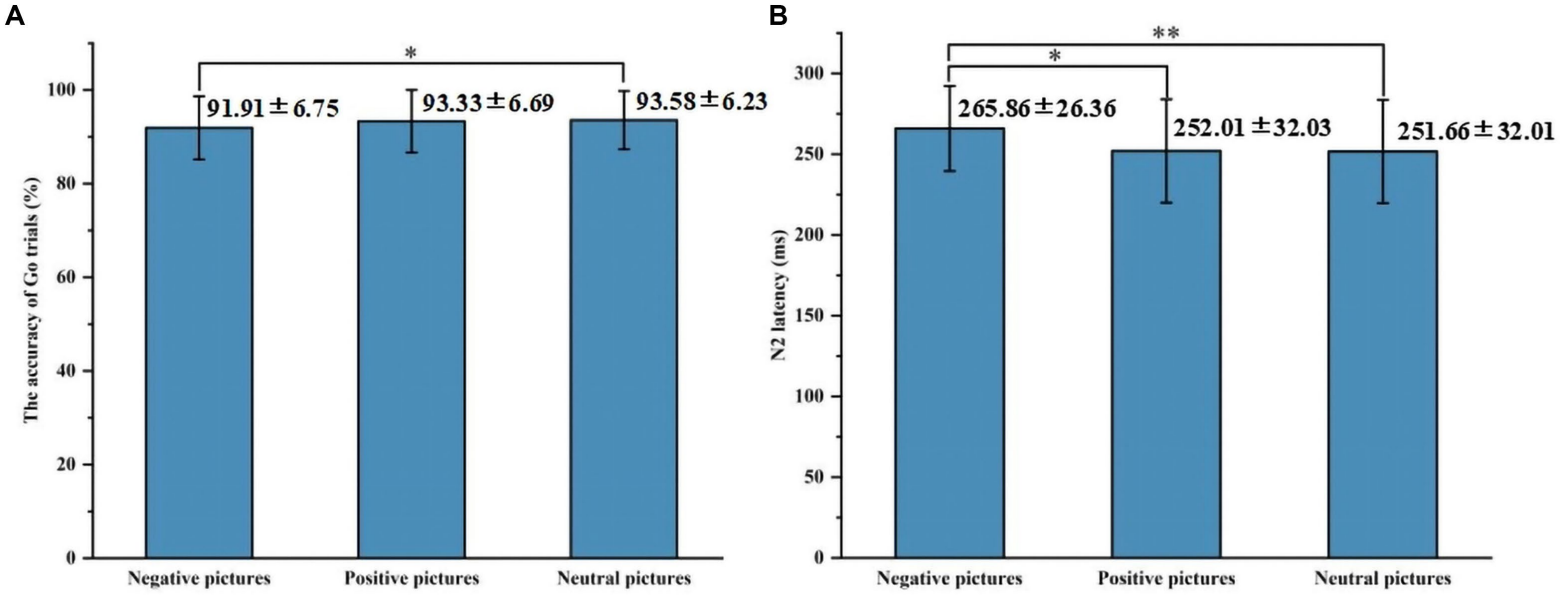
The results of the accuracy of Go trials and N2 latency in Experiment 1. **(A)** Comparison of the accuracy of Go trials under three emotional conditions. **(B)** Comparison of N2 latency under three emotional conditions. *p < 0.05, **p < 0.01.

Behavioral results are summarized in Table 2. In the accuracy of Go trials, the main effect of emotional valence was statistically significant (*F* = 4.297, *p* = 0.017), and the accuracy of Go trials under negative conditions was lower than that under neutral conditions (*p* = 0.020). There was no significant difference between the accuracy under negative conditions and that under positive conditions (*p* = 0.090), and the accuracy under positive conditions and that under neutral conditions (*p* = 1.000), as shown in Figure 2. In the accuracy of Stop trials, SSD, and SSRT, there was no significant difference in the main effect of group, the main effect of emotional valence, and the interaction between group and valence.

**Table 2:** Behavioral results on stop signal task in Experiment 1 (M ± SD).

Emotional types	The health group (*n* = 18)	The MCI group (*n* = 18)	The main effect		The interaction between group and valence
			Group	Valence	
The accuracy of Go trials (%)					
Negative pictures	90.74 ± 6.87	93.09 ± 6.62	*F* = 2.66	*F* = 4.30*	*F* = 0.94
Positive pictures	91.54 ± 6.86	95.12 ± 6.20			
Neutral pictures	91.61 ± 6.92	95.56 ± 4.88			
The accuracy of Stop trials (%)					
Negative pictures	55.00 ± 9.24	50.74 ± 12.24	*F* = 1.08	*F* = 1.68	*F* = 0.24
Positive pictures	53.70 ± 8.99	49.81 ± 13.26			
Neutral pictures	54.44 ± 10.67	51.11 ± 12.58			
SSD (ms)					
Negative pictures	361.67 ± 149.60	284.07 ± 142.83	F = 2.66	*F* = 1.76	*F* = 0.25
Positive pictures	352.41 ± 155.66	275.74 ± 132.43			
Neutral pictures	367.22 ± 165.67	282.59 ± 138.84			
SSRT (ms)					
Negative pictures	265.26 ± 46.34	280.43 ± 52.24	*F* = 1.32	*F* = 0.52	*F* = 0.26
Positive pictures	272.35 ± 41.48	285.20 ± 38.35			
Neutral pictures	261.61 ± 55.50	283.88 ± 60.08			

SSD, Stop Signal Delay; SSRT, Stop Signal Reaction Time. *p < 0.05.

#### 3.1.3 ERPs results

**Figure 3:**
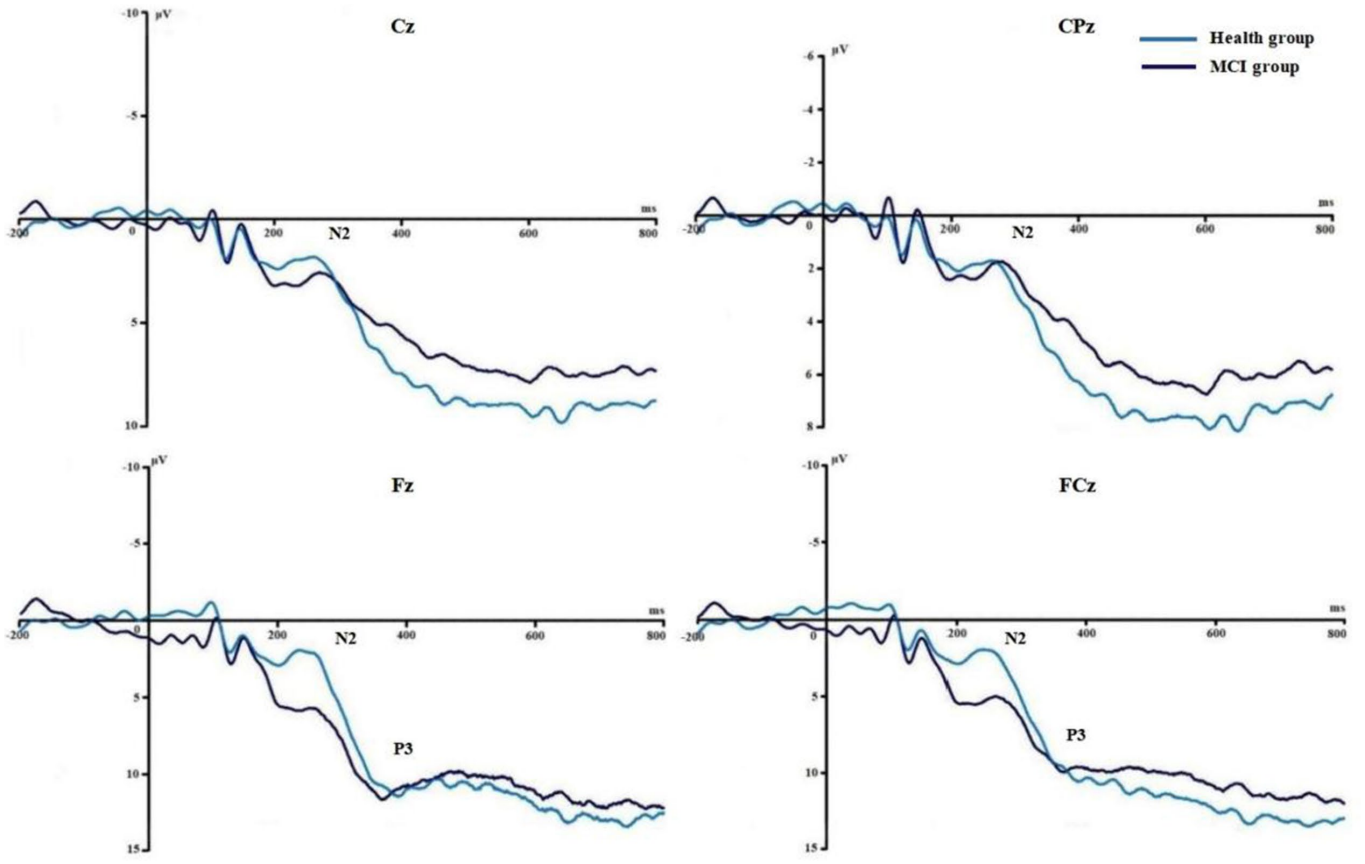
Comparison of N2 and P3 between the two groups in Experiment 1.

**Figure 4:**
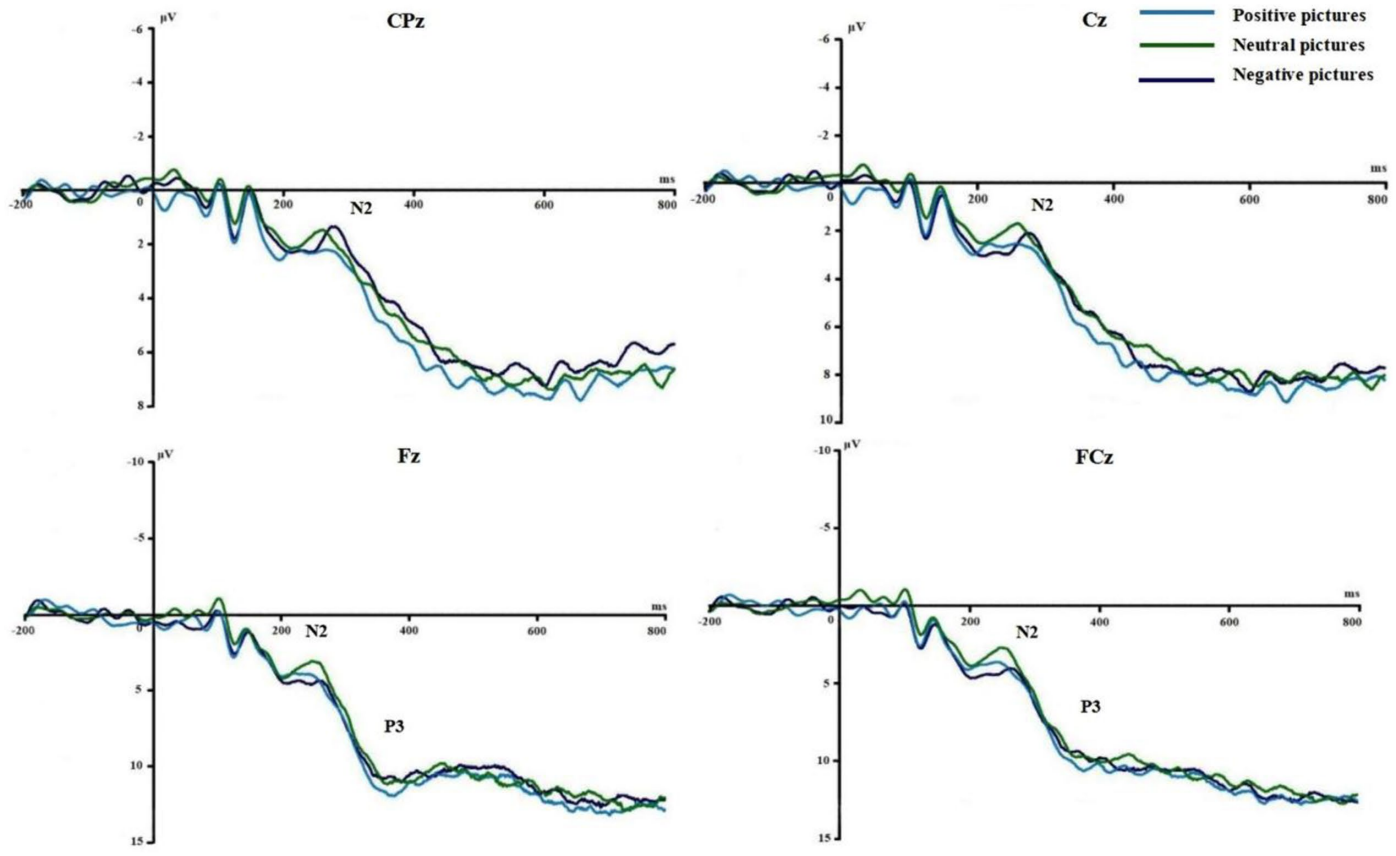
Comparison of N2 and P3 under different emotions in Experiment 1.

Figures 3, 4 show the grand average ERPs of stop signals in two groups and three emotional conditions. The main effect of emotional valence in N2 latency was statistically significant (*F* = 5.881, *p* = 0.004), and the N2 latency in the negative condition was significantly greater than that in the positive (*p* = 0.032) and neutral (*p* = 0.008) conditions, as shown in Figure 2. There was no significant difference in N2 latency between positive and neutral conditions (*p* = 1.000). In N2 amplitude, P3 amplitude and latency, there was no significant difference in the main effect of group, the main effect of emotional valence, and the interaction between group and valence, as shown in Table 3.

**Table 3:** ERPs results on stop signal task in Experiment 1 (M ± SD).

Emotional types	The health group (*n* = 18)	The MCI group (*n* = 18)	The main effect		The interaction between group and valence
			Group	Valence	
N2 amplitude (μV)					
Negative pictures	−0.08 ± 5.05	1.94 ± 4.69	*F* = 2.84	*F* = 0.39	*F* = 0.66
Positive pictures	0.082 ± 3.92	1.65 ± 4.51			
Neutral pictures	−0.98 ± 4.94	1.90 ± 5.05			
N2 latency (ms)					
Negative pictures	259.26 ± 23.43	272.46 ± 27.61	*F* = 1.02	F = 5.88**	*F* = 0.62
Positive pictures	248.76 ± 33.35	255.25 ± 30.54			
Neutral pictures	250.24 ± 30.92	253.08 ± 33.21			
P3 amplitude (μV)					
Negative pictures	11.67 ± 6.34	9.75 ± 7.16	F = 0.25	*F* = 1.27	*F* = 1.75
Positive pictures	11.55 ± 6.10	11.27 ± 7.39			
Neutral pictures	11.04 ± 5.52	10.56 ± 6.24			
P3 latency (ms)					
Negative pictures	394.39 ± 50.00	405.03 ± 43.71	*F* = 0.00	*F* = 0.78	*F* = 1.43
Positive pictures	403.07 ± 43.94	405.11 ± 42.53			
Neutral pictures	401.92 ± 42.95	387.58 ± 46.06			

^**^p < 0.01.

#### 3.1.4 Correlations between sociodemographic and neuropsychological data and ERPs measurements

It was found that there was a significant positive correlation between years of education and MoCA total score (*r* = 0.418, *p* = 0.011). SSRT was significantly correlated with P3 amplitude (*r* = −0.387, *p* = 0.020) and latency (*r* = 0.353, *p* = 0.035), indicating that the greater the SSRT was, the smaller the P3 amplitude was, the longer the P3 latency was, and the worse the response inhibition was (see Supplementary Table 1).

### 3.2 Experiment 2

#### 3.2.1 Participant characteristics

Among the 44 participants, 7 were excluded because the collected ERP data had too many bad segments and could not be used, and 37 participants were eventually included, 19 in the MCI group and 18 in the health group. As can be seen in Table 4, the two groups did not differ in age, gender and years of education. The total score of MoCA in the MCI group was lower than that in the health group (Z = −4.600, *p* < 0.001), and the cognitive function of the MCI group was decreased to different degrees, which were manifested in Visuospatial/Executive (*p* = 0.033), Naming (*p* < 0.001), Attention (*p* = 0.019), Language (*p* = 0.026), and Delayed recall (*p* = 0.005).

**Table 4:** Sociodemographic and neuropsychological data for two groups in Experiment 2.

	The health group (*n* = 18)	The MCI group (*n* = 19)	χ^2^ /Z	*p*
Gender (Male / Female)	6 / 12	5 / 14	0.22	0.641
Age	56.00 (52.00, 64.75)	59.00(57.00, 61.00)	−1.48	0.139
Education (years)	9.00 (6.75, 11.00)	8.00 (7.00, 9.00)	−0.79	0.430
The total score of MoCA	25.00 (25.00, 27.25)	21.00 (19.00, 23.00)	−4.60	**<0.001**
Visuospatial/Executive	4.00 (3.00, 5.00)	3.00 (2.00, 4.00)	−2.14	**0.033**
Naming	2.50 (2.00, 3.00)	2.00 (1.00, 2.00)	−3.53	**<0.001**
Attention	6.00 (5.00, 6.00)	5.00 (4.00, 6.00)	−2.35	**0.019**
Language	3.00 (2.00, 3.00)	2.00 (2.00, 3.00)	−2.23	**0.026**
Abstraction	1.00 (0.00, 2.00)	0.00 (0.00, 1.00)	−1.55	0.120
Delayed recall	3.00 (2.00, 4.00)	2.00 (1.00, 2.00)	−2.83	**0.005**
Orientation	6.00 (6.00, 6.00)	6.00 (5.00, 6.00)	−1.16	0.244
TMT-A (errors)	0.00 (0.00, 0.00)	0.00 (0.00, 0.00)	−0.90	0.367
TMT-A (s)	54.50 (36.75, 78.25)	59.00 (46.00, 76.00)	−0.61	0.543

MoCA, the Montreal Cognitive Assessment; TMT-A, Trail Making Test A. All significant *p* values are shown in bold.

#### 3.2.2 Behavioral results

**Figure 5:**
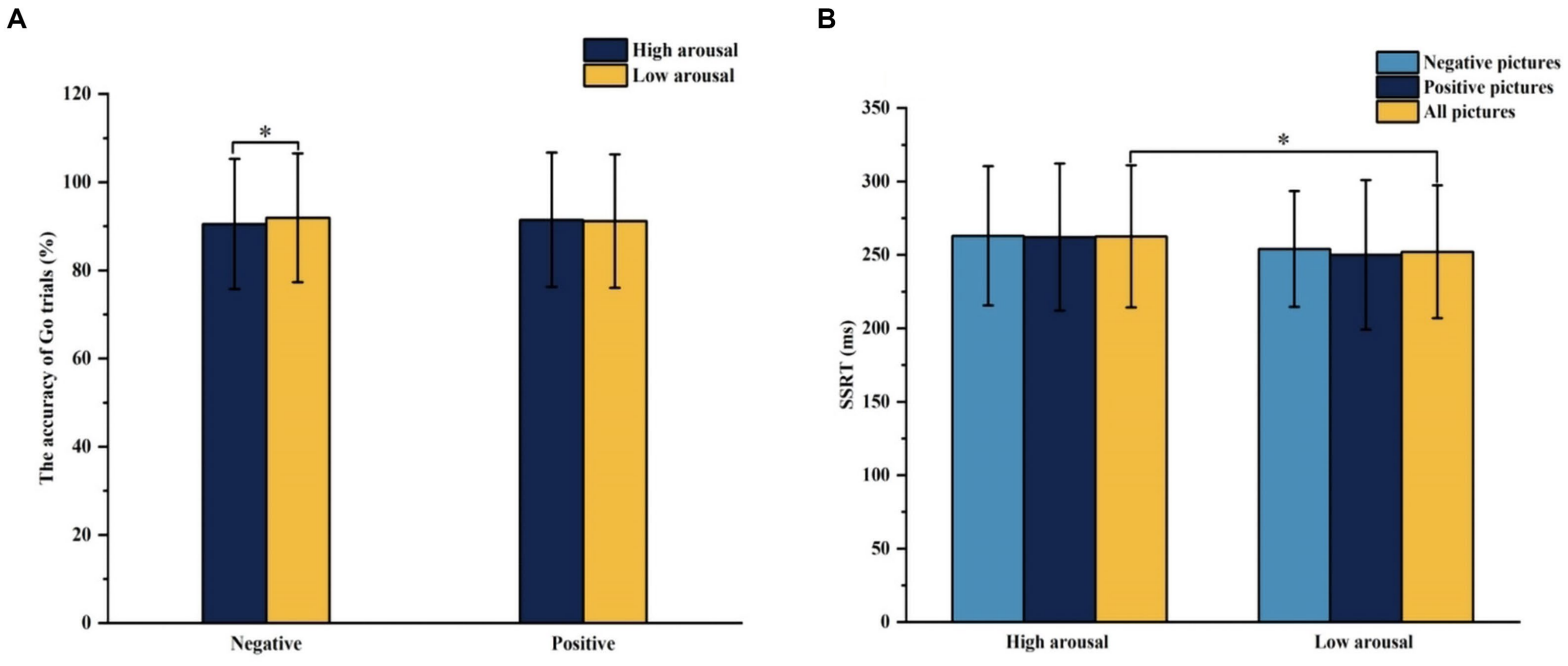
The results of the accuracy of Go trials and SSRT under different emotional conditions in Experiment 2. **(A)** Comparison of the accuracy of Go trials under different emotional valence and arousal. **(B)** Comparison of SSRT under different emotional valence and arousal. *p < 0.05, SSRT, Stop Signal Reaction Time.

The interaction effect between emotional valence and arousal for Go trials accuracy was statistically significant (*F* = 5.398, *p* = 0.026), and the simple effect of arousal was statistically significant in negative conditions (*F* = 6.770, *p* = 0.013). The accuracy of Go trials under high arousal negative conditions was lower than that under low arousal negative conditions. In the stop trails, the main effect of emotional arousal in the SSRT was significant (*F* = 4.790, *p* = 0.035) and the SSRT of high arousal emotional pictures (262.521 ms) was significantly greater than that of low arousal emotional pictures (251.979 ms), as shown in Figure 5. In the accuracy of Stop trials and SSD, there was no significant difference in the main effect of group, the main effect of emotional valence, the main effect of emotional arousal, and the interaction of group, valence, and arousal, as shown in Table 5.

**Table 5:** Behavioral results on stop signal task in Experiment 2 (M ± SD).

	The health group (*n* = 18)	The MCI group (*n* = 19)	The main effect			The interaction effect			
			Group	Valence	Arousal	Group× Valence	Group× Arousal	Valence× Arousal	Group×Valence×Arousal
The accuracy of go trials (%)									
High arousal positive pictures	92.59 ± 7.36	90.41 ± 20.31	*F* = 0.19	*F* = 0.08	*F* = 2.63	*F* = 0.01	*F* = 0.02	*F* = 5.40*	F = 0.02
High arousal negative pictures	91.54 ± 7.06	89.53 ± 19.68							
Low arousal positive pictures	92.28 ± 6.45	90.12 ± 20.36							
Low arousal negative pictures	93.03 ± 6.85	90.82 ± 19.47							
The accuracy of stop trials (%)									
High arousal positive pictures	55.00 ± 11.16	53.69 ± 19.37	*F* = 0.10	*F* = 0.51	F = 0.01	*F* = 0.17	*F* = 0.11	F = 0.02	*F* = 0.37
High arousal negative pictures	55.93 ± 12.66	53.68 ± 18.08							
Low arousal positive pictures	55.19 ± 11.45	53.69 ± 20.09							
Low arousal negative pictures	55.56 ± 11.60	54.04 ± 17.13							
SSD (ms)									
High arousal positive pictures	400.37 ± 156.85	374.21 ± 189.08	F = 0.26	*F* = 3.37	*F* = 3.36	*F* = 0.09	*F* = 0.04	*F* = 0.06	*F* = 0.35
High arousal negative pictures	395.00 ± 151.18	367.10 ± 180.95							
Low arousal positive pictures	412.87 ± 153.05	380.70 ± 183.58							
Low arousal negative pictures	401.76 ± 145.46	375.96 ± 177.84							
SSRT (ms)									
High arousal positive pictures	260.58 ± 43.20	263.68 ± 56.93	F = 0.24	F = 0.52	F = 4.79*	F = 0.19	F = 0.04	F = 0.19	F = 0.11
High arousal negative pictures	258.65 ± 28.78	267.17 ± 60.69							
Low arousal positive pictures	246.16 ± 40.62	253.72 ± 59.87							
Low arousal negative pictures	249.99 ± 31.74	258.04 ± 46.04							

SSD, Stop Signal Delay; SSRT, Stop Signal Reaction Time. *p < 0.05.

#### 3.2.3 ERPs results

**Figure 6:**
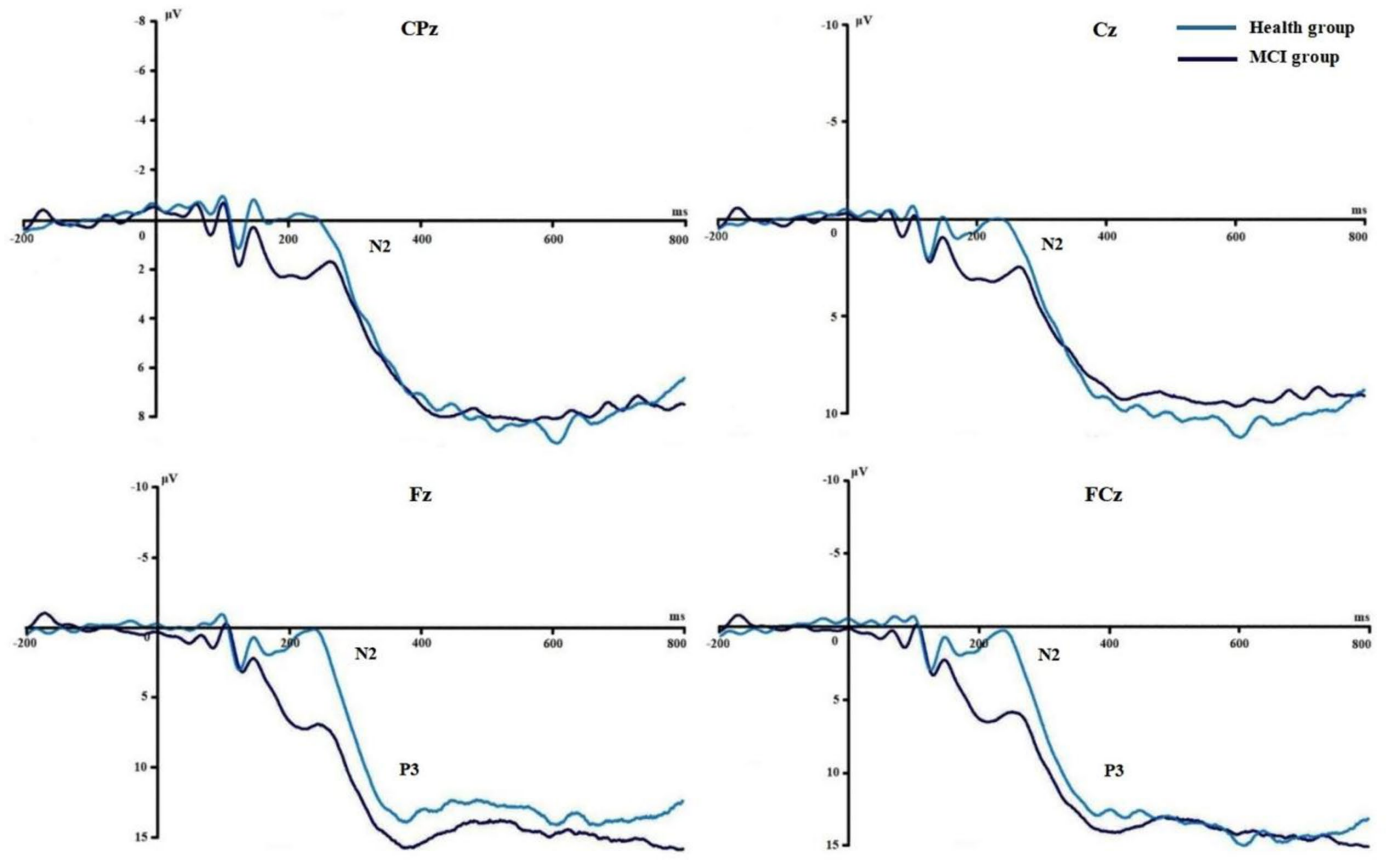
Comparison of N2 and P3 between the two groups in Experiment 2.

**Figure 7:**
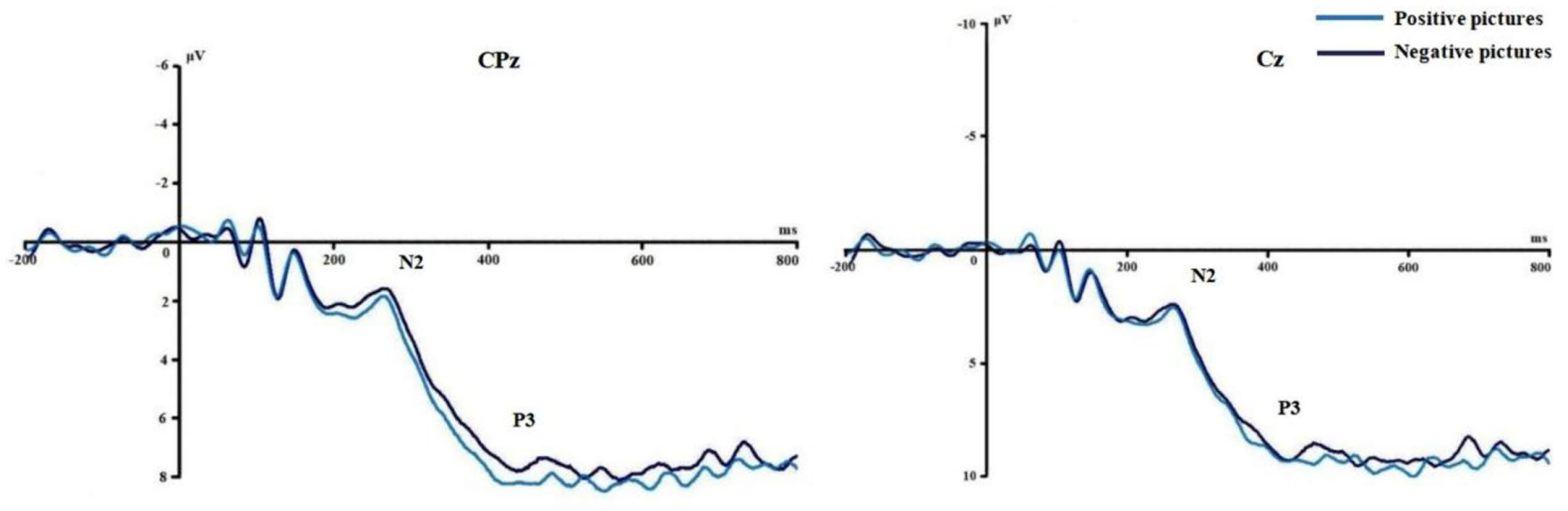
Comparison of N2 and P3 between the two emotional valence in MCI in Experiment 2.

**Figure 8:**
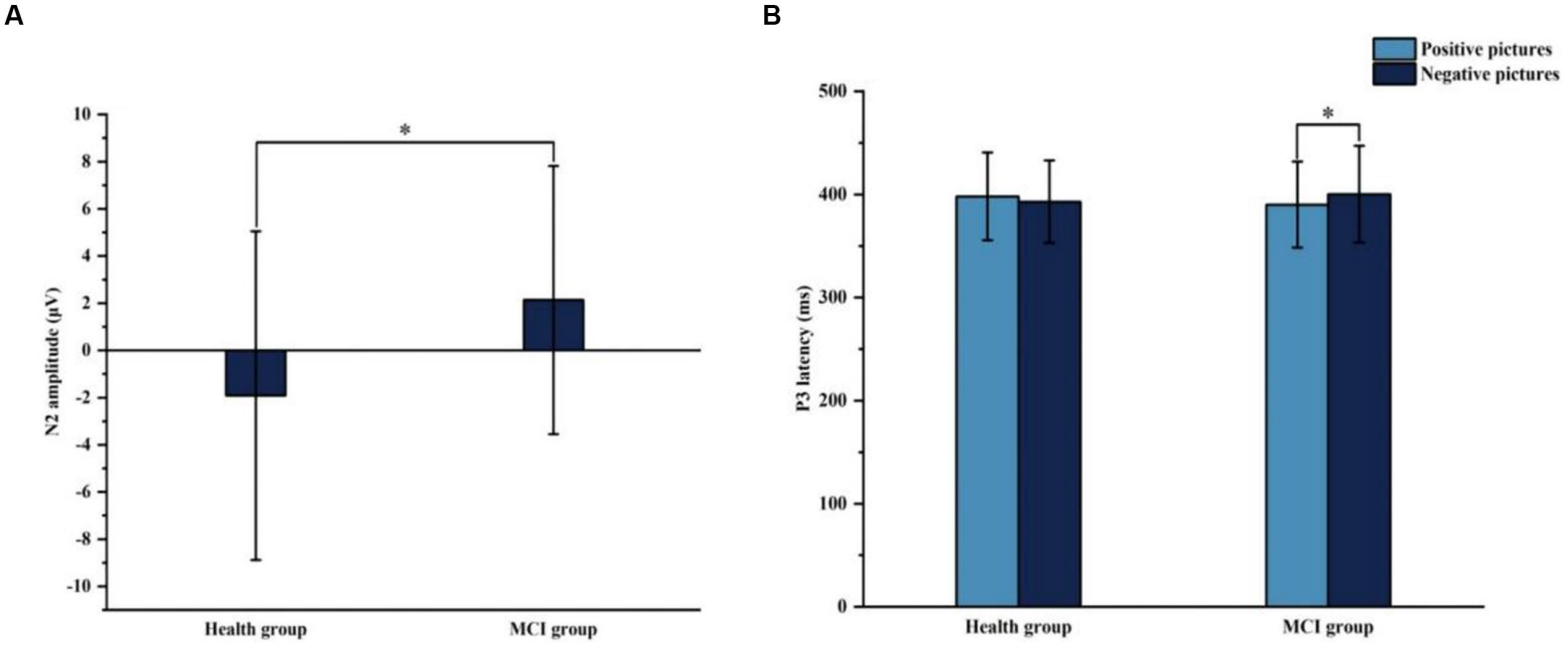
The results of N2 amplitude and P3 latency in Experiment 2. **(A)** Comparison of N2 amplitude in two groups. **(B)** Comparison of P3 latency in two groups with different emotion valence. *p < 0.05.

Figures 6, 7 show the grand average ERPs of stop signals in two groups and two emotional valence conditions. The main effect of group in N2 amplitude was statistically significant (*F* = 4.860, *p* = 0.034), and N2 amplitude in the MCI group (2.136 μV) was smaller than that in the health group (−1.914 μV). The interaction effect between group and valence in P3 latency was statistically significant (*F* = 5.091, *p* = 0.030), and the simple effect of valence in MCI group was statistically significant (*F* = 4.644, *p* = 0.038). The P3 latency under negative pictures (400.276 ms) was greater than that under positive pictures (390.132 ms), as shown in Figure 8. In N2 latency and P3 amplitude, there was no significant difference in the main effect of group, the main effect of emotional valence, the main effect of emotional arousal, and the interaction of group, valence, and arousal, as shown in Table 6.

**Table 6:** ERPs results on stop signal task in Experiment 2 (M ± SD).

	The health group (*n* = 18)	The MCI group (*n* = 19)	The main effect			The interaction effect			
			Group	Valence	Arousal	Group× Valence	Group× Arousal	Valence× Arousal	Group×Valence×Arousal
N2 amplitude (μV)									
High arousal positive pictures	−2.20 ± 6.40	2.72 ± 5.27	F = 4.86*	F = 0.02	*F* = 3.12	*F* = 0.07	F = 0.09	*F* = 0.56	*F* = 3.54
High arousal negative pictures	−0.81 ± 8.49	2.14 ± 6.74							
Low arousal positive pictures	−1.80 ± 5.47	1.61 ± 5.52							
Low arousal negative pictures	−2.85 ± 7.15	2.08 ± 5.14							
N2 latency (ms)									
High arousal positive pictures	239.58 ± 30.57	242.57 ± 25.77	F = 0.00	*F* = 1.95	*F* = 0.49	F = 0.35	F = 0.26	*F* = 0.45	*F* = 1.17
High arousal negative pictures	242.75 ± 20.22	242.51 ± 29.01							
Low arousal positive pictures	240.22 ± 25.98	234.25 ± 29.27							
Low arousal negative pictures	241.04 ± 24.54	244.20 ± 28.84							
P3 amplitude (μV)									
High arousal positive pictures	13.30 ± 8.36	14.37 ± 7.38	F = 0.14	*F* = 0.05	*F* = 0.46	*F* = 0.16	*F* = 0.86	*F* = 0.30	F = 0.10
High arousal negative pictures	13.43 ± 8.38	14.52 ± 7.09							
Low arousal positive pictures	13.73 ± 7.37	13.91 ± 7.48							
Low arousal negative pictures	13.17 ± 7.93	13.88 ± 6.49							
P3 latency (ms)									
High arousal positive pictures	394.58 ± 41.58	392.37 ± 37.42	F = 0.00	F = 0.56	*F* = 0.50	F = 5.09*	F = 0.05	*F* = 0.12	*F* = 2.27
High arousal negative pictures	394.65 ± 41.46	394.36 ± 49.46							
Low arousal positive pictures	401.64 ± 43.69	387.90 ± 45.87							
Low arousal negative pictures	391.40 ± 38.56	406.20 ± 43.64							

*p < 0.05.

#### 3.2.4 Correlations between sociodemographic and neuropsychological data and ERP measurements

Years of education was positively correlated with the MoCA total score (*r* = 0.469, *p* = 0.003), similar to the results of Experiment 1 (see Supplementary Table 2). SSRT was positively correlated with P3 latency (*r* = 0.376, *p* = 0.022), indicating that the greater the SSRT was, the longer the P3 latency was. When MCI does not show whether their response inhibition is impaired in behavioral manifestations, ERP can be used to early detect response inhibition.

## 4 Discussion

### 4.1 Effects of emotional valence on response inhibition

The results showed that emotional valence interferes with response inhibition. Specifically, the N2 latency under negative conditions was significantly greater than that under positive and neutral conditions. Previous research has shown that N2 reflects decision-making and conflict monitoring (López Zunini et al., 2016), indicating that negative stimuli could lead to decision-making and conflict monitoring delays. Moreover, in MCI, the P3 latency under negative conditions was significantly longer than that under positive conditions, which indicated that negative stimulation caused a delay in participants’ response inhibition. The dual competition framework suggests that when emotional information is not task-related, performance impairment by emotional information is usually observed because resources are taken away from the main task (Pessoa, 2009). Emotional experiences interfere with the effectiveness of inhibitory control by capturing attention automatically and guiding behavior. More specifically, negative emotions capture more attention resources than positive emotions, thus interfering more with response inhibition. Protecting the self may drive the prioritization of negative stimulus processing (Rebetez et al., 2015). Carretié et al. examined attentional habituation in response to emotional stimuli and found that negative emotions were more resistant to habituation, which reflected the greater capacity of negative emotions to attract and maintain the participant’s attention (Carretié et al., 2003). The other study has shown that performing the stop-signal task after presenting negative stimuli reduced activation of the dorsolateral prefrontal cortex, medial frontal cortex, ventrolateral prefrontal cortex, and parietal cortex and decreased neural connectivity. Negative emotions can prospectively impair response inhibition through a mechanism other than attentional capture, representing a modulated change in the internal state resulting from top-down processes (Patterson et al., 2016).

### 4.2 Effects of emotional arousal on response inhibition

SSRT is the most critical indicator in the stop-signal task. The lower the SSRT, the shorter the reaction time to the stop-signal, and the quicker they can suppress response impulses. The results of Experiment 2 showed that the SSRT of high-arousal pictures was significantly greater than the SSRT of low-arousal-pictures, consistent with the previous study that the high-arousal pictures impaired the response inhibition (Yu et al., 2015; Zhao et al., 2019). Zhao et al. found that smaller N2 amplitudes for Nogo faces in the negative and positive conditions than in the neutral condition, suggesting response inhibition is influenced by emotion arousal and emotion conditions reduce attention allocation during conflict monitoring of Nogo trials (Zhao et al., 2019). The possible reason is that prioritized processing of high-arousal information results in greater attention being captured, thereby impairing other executive functions that rely on common-pool resources, including inhibition, shifting, and updating. The impairment will be typically observed when the high-arousal information is task-irrelevant (Pessoa, 2009). Arousal-biased competition theory suggests that high-arousal stimuli gain the competitive advantage and that this prioritized processing of arousing images is due to increased amygdala activation during the processing of these stimuli and the influence of amygdala activation on frontoparietal attention networks (Singh and Sunny, 2017). In addition, the results showed that the accuracy of Go trials under high-arousal negative pictures was significantly lower than that under low-arousal negative pictures. High-arousal stimuli elicited greater interference than low-arousal stimuli, and the effect was more pronounced in the negative than in the positive condition (Compton et al., 2003). German word ratings show that an increase in arousal often accompanies the increase in negative valence, that negative information has high arousal, and that the correlation between valence and arousal appears significantly weakened for positive words (Recio et al., 2014). Negative information is more likely to be associated with high arousal than positive information (Lang et al., 1997). Hofmann et al. concluded that emotional arousal appears to have a moderating effect on emotional valence, which was mainly reflected in modulating behavioral responses to negative words (Hofmann et al., 2009), determining whether the effect of negative valence was found or not and affecting an early allocation of attention and the preferential processing (Vieitez et al., 2021). In summary, it is necessary to explore effective emotion regulation strategies to improve the emotional state and weaken the damage of emotion to response inhibition.

### 4.3 Comparison of response inhibition in MCI and health groups

Previous research has shown that despite age-related declines in cognitive control and inhibition of responses to non-emotional information, older adults may use their emotion regulation abilities to show better emotional response inhibition than younger adults (Waring et al., 2019). Since more and more older adults have pathological cognitive function impairment, this study further explored the emotional response inhibition of MCI, which is at the initial stage of cognitive function impairment. The significant correlation between SSRT and ERP components suggested that when the impairment of response inhibition in MCI was not sufficiently manifested in behavior, early ERP monitoring can identify neuro-electrophysiological indicators and the time course of impairment of response inhibition in MCI. ERP results showed that N2 amplitude was significantly smaller in the MCI group than in the health group, suggesting deficits in response conflict monitoring and inhibitory processes in the MCI group, and the results support previous findings of impaired inhibitory control in MCI (Cid-Fernández et al., 2014, 2017). The smaller amplitude of the N2 in the MCI group may reflect deficits in neural networks responsible for inhibitory control, such as the anterior cingulate cortex, the inferior frontal, and the orbitofrontal cortices (Cid-Fernández et al., 2014). Patients with depression or anxiety similarly show impaired response inhibition (Pacheco-Unguetti et al., 2010; García-Martín et al., 2021). Depression and anxiety are common in MCI (Ismail et al., 2017). The prevalence of depression in patients with MCI in community-based samples was 25% and was 40% in clinic-based samples (Ismail et al., 2017). Depressive and anxiety symptoms determine an additive risk effect to the progression to dementia in people with MCI (Palmer et al., 2007; Mourao et al., 2016). 27.5% of participants with depression at baseline developed dementia, compared with 14.8% of those without depression (Chan et al., 2011). 83.3% of those with MCI and anxiety symptoms developed Alzheimer’s disease, and 40.9% of those with MCI but no anxiety symptoms (Palmer et al., 2007). Increased anxiety symptoms may be associated with poorer global cognition, episodic memory, and executive functioning (Beaudreau and O'Hara, 2008). Anxiety and depressive symptoms in MCI may be related to their impaired response inhibition.

In addition, depression and anxiety were both significantly and positively correlated with maladaptive emotion regulation strategies, including avoidance and rumination (Schäfer et al., 2017). MCI may tend to use negative cognitive emotion regulation strategies, and the elderly were more accustomed to using emotion regulation strategies such as expressive suppression (Eldesouky and English, 2018), which may not reduce the experience of negative emotions and inhibit the expression of positive emotions. Consequently, improving inappropriate emotion regulation strategies is critical to reducing the impact of emotions on response inhibition in MCI. The results of correlation analysis showed that the years of education are positively correlated with the MoCA score. There was a correlation between the low level of education and low total MoCA scores. The low total MoCA scores may have been due to the participants’ low cognitive function, or the low level of education made it difficult for the participants to comprehend the task requirements of the MoCA scales, or they have less experience with the MoCA scales. Previous research found that people with low education showed poor cognitive performance (Barbosa et al., 2021; Kowall and Rathmann, 2023). More years of education could increase the memory score four decades later and also have a protective effect of schooling on cognitive decline in terms of verbal fluency (Schneeweis et al., 2014). It is recommended that follow-up research combine emotion-cognitive regulation strategies with educational intervention to investigate the effects on the response inhibition in MCI. Such intervention experiments could use neuropsychological assessments and other non-invasive methods, such as ERPs, to explore the intervention effect.

Readers must be aware of several limitations of this study. The first limitation is that this study’s arousal of emotional materials consisted of only two levels, high and low. However, some studies have analyzed emotional arousal at multiple levels, such as three levels, including high, medium, and low. Secondly, the present study did not consider the analysis of effect sizes. Thirdly, depression and anxiety were not measured. Future relevant research could compensate for these limitations to more fully examine the impact of emotions on inhibitory control in MCI.

## Data availability statement

The original contributions presented in the study are included in the article/Supplementary material, further inquiries can be directed to the corresponding author.

## Ethics statement

The studies involving humans were approved by the Ethics Committee of Nanjing Medical University and the number of the ethical committee was (2021) No. 553. The studies were conducted in accordance with the local legislation and institutional requirements. The participants provided their written informed consent to participate in this study.

## Author contributions

JW: Conceptualization, Formal analysis, Visualization, Writing – original draft, Writing – review & editing, Data curation. CL: Conceptualization, Methodology, Writing – original draft, Writing – review & editing, Data curation. XY: Conceptualization, Investigation, Visualization, Writing – review & editing. YZ: Conceptualization, Investigation, Visualization, Writing – review & editing. ES: Methodology, Project administration, Supervision, Writing – review & editing. YX: Methodology, Project administration, Supervision, Writing – review & editing. XL: Conceptualization, Funding acquisition, Project administration, Resources, Supervision, Writing – review & editing.
