# Supplementary material for: Effect of emotional stimulus on response inhibition in people with mild cognitive impairment: an event-related potential study
Source: Front Neurosci. 2024 Apr 30;18:1357435. doi: 10.3389/fnins.2024.1357435 (PMC11091389; doi:10.3389/fnins.2024.1357435)
Supplement: Supplementary file 1 [file Data_Sheet_1.docx]

Supplementary Material

**Effect of emotional stimulus on response inhibition in people with mild cognitive impairment: An event-related potential study**

**Jing Wang ^†^, Cheng Li ^†^, Xiaohong Yu, Yue Zhao, Enfang Shan, Ying Xing, Xianwen Li ^*^**

†Jing Wang and Cheng Li contributed equally to this work and shared first authorship.

*** Correspondence:** Xianwen Li, E-mail address: [xwli0201@njmu.edu.cn](mailto:xwli0201@njmu.edu.cn)

**Supplementary Table 1.** Correlations between Sociodemographic and neuropsychological data and ERP measurements in Experiment 1

|  | **Age** | **Years of education** | **MoCA** | **The accuracy of Go trials** | **SSRT** | **N2 amplitude** | **N2 latency** | **P3 amplitude** |
| --- | --- | --- | --- | --- | --- | --- | --- | --- |
| **Years of education** | 0.316 |  |  |  |  |  |  |  |
| **MoCA** | -0.212 | 0.418 * |  |  |  |  |  |  |
| **The accuracy of Go trials** | -0.042 | -0.141 | -0.174 |  |  |  |  |  |
| **SSRT** | 0.060 | -0.032 | -0.280 | 0.134 |  |  |  |  |
| **N2 amplitude** | 0.199 | 0.196 | -0.131 | 0.120 | -0.116 |  |  |  |
| **N2 latency** | 0.039 | 0.025 | -0.018 | -0.075 | 0.169 | -0.066 |  |  |
| **P3 amplitude** | 0.105 | 0.108 | 0.133 | 0.056 | -0.387 * | 0.403 * | -0.161 |  |
| **P3 latency** | 0.157 | -0.080 | 0.106 | 0.072 | 0.353 * | -0.479 ** | 0.321 | -0.263 |
| ***P＜0.05; **P＜0.01** | | | | | | | | |

**Supplementary Table 2.** Correlations between Sociodemographic and neuropsychological data and ERP measurements in Experiment 2

|  | **Age** | **Years of education** | **MoCA** | **The accuracy of Go trials** | **SSRT** | **N2 amplitude** | **N2 latency** | **P3 amplitude** |
| --- | --- | --- | --- | --- | --- | --- | --- | --- |
| **Years of education** | 0.123 |  |  |  |  |  |  |  |
| **MoCA** | -0.268 | 0.469 ** |  |  |  |  |  |  |
| **The accuracy of Go trials** | -0.018 | 0.092 | 0.008 |  |  |  |  |  |
| **SSRT** | 0.208 | 0.061 | -0.218 | -0.015 |  |  |  |  |
| **N2 amplitude** | 0.051 | 0.282 | -0.099 | -0.096 | 0.023 |  |  |  |
| **N2 latency** | 0.076 | -0.093 | 0.069 | 0.038 | 0.222 | -0.331 * |  |  |
| **P3 amplitude** | -0.070 | 0.098 | 0.064 | -0.059 | -0.284 | 0.577 ** | -0.351 * |  |
| **P3 latency** | 0.276 | -0.140 | -0.025 | -0.053 | 0.376 * | -0.495 ** | 0.534 ** | -0.271 |
| ***P＜0.05; **P＜0.01** | | | | | | | | |
